# Injection drug network characteristics as a predictor of injection behaviour

**DOI:** 10.1017/S095026881900061X

**Published:** 2019-04-05

**Authors:** Tim Spelman, Rachel Sacks-Davis, Paul Dietze, Peter Higgs, Margaret Hellard

**Affiliations:** 1 Burnet Institute, Melbourne, Australia; 2 Department of Epidemiology and Preventive Medicine, Monash University, Melbourne, Australia; 3 Department of Public Health, School of Psychology & Public Health, La Trobe University, Melbourne, Australia; 4 Department of Infectious Disease, Alfred Hospital, Melbourne, Australia

**Keywords:** Hepatitis C, injecting drug-users (IDUs), surveillance, transmission

## Abstract

Social network characteristics of people who inject drugs (PWID) have previously been flagged as potential risk factors for HCV transmission such as increased injection frequency. To understand the role of the injecting network on injection frequency, we investigated how changes in an injecting network over time can modulate injecting risk behaviour. PWID were sourced from the Networks 2 Study, a longitudinal cohort study of PWID recruited from illicit drug street markets across Melbourne, Australia. Network-related correlates of injection frequency and the change in frequency over time were analysed using adjusted Cox Proportional Hazards and Generalised Estimating Equations modelling. Two-hundred and eighteen PWID followed up for a mean (s.d.) of 2.8 (1.7) years were included in the analysis. A greater number of injecting partners, network closeness centrality and eigenvector centrality over time were associated with an increased rate of infection frequency. Every additional injection drug partner was associated with an increase in monthly injection frequency. Similarly, increased network connectivity and centrality over time was also associated with an increase in injection frequency. This study observed that baseline network measures of connectivity and centrality may be associated with changes in injection frequency and, by extension, may predict subsequent HCV transmission risk. Longitudinal changes in network position were observed to correlate with changes in injection frequency, with PWID who migrate from the densely-connected network centre out to the less-connected periphery were associated with a decreased rate of injection frequency.

## Introduction

Behavioural and demographic correlates of injection frequency through injection drug networks have been well documented [1–18]. People who inject drugs (PWID) commonly function within a wider social network of other PWID [19]. The number of injecting partners and how central a person is within such a network have previously been flagged as potential predictors of pathogen transmission [19, 20]. Increasing the size of the injection drug network and density has been reported to correlate with an increased frequency of risky injecting behaviours that may in turn increase HCV exposure risk [21–25]. Previous longitudinal models of HCV transmission in PWID have largely presumed a homogenous population, where any one PWID is equally likely to have contact with all other PWID within an injection drug network (i.e. the full mixing model assumption) [26]. However, recent studies have observed that injecting networks are typically characterised by a high degree of heterogeneity [27–30]. This may translate into a range of risk patterns sub-populations within a network, which in turn may impact on the risk of HCV transmission through the network [18, 27].

Network sub-populations of PWID linked by drug use preferences within an injecting network have previously been observed to correlate with HBV and HCV transmission [28]. Similarly drug-using PWID network sub-populations have previously been observed to correlate with higher degrees of syringe-sharing, relative to other non-drug-using segments of the network [30]. Furthermore, a PWID's location within an injection drug network may predict subsequent exposure to HCV [29]. Rolls *et al*. simulated HCV transmission over an empirical social PWID network and observed that HCV incidence varied with the number of injecting partners in a PWID's immediate personal network [31]. Increasing network turnover has also been observed to correlate with an increase in injection risk behaviours [32], whilst the influence of more active subsets of injectors within the network on facilitating the transition of non-injecting heroin users to active injectors has previously been observed [32]. PWID centrality within an injection drug network can further moderate risk behaviour [29, 33, 34].

Much of the existing research into the role of network structure on infection risk has focused on HIV, whilst much of the evidence around network correlates of, specifically, HCV infection risk has been based on single time-point and cross-sectional data. Whilst baseline network correlates of HCV transmission risk have previously been studied, this is the first study to investigate how longitudinal changes in the structure of an injecting network over time modulate injecting risk behaviour and HCV transmission risk. The objective of this study was to examine baseline and time-varying injection drug network characteristics as correlates of sustained changes in injection frequency over time.

## Materials and methods

### Participants

**Fig. 1.**
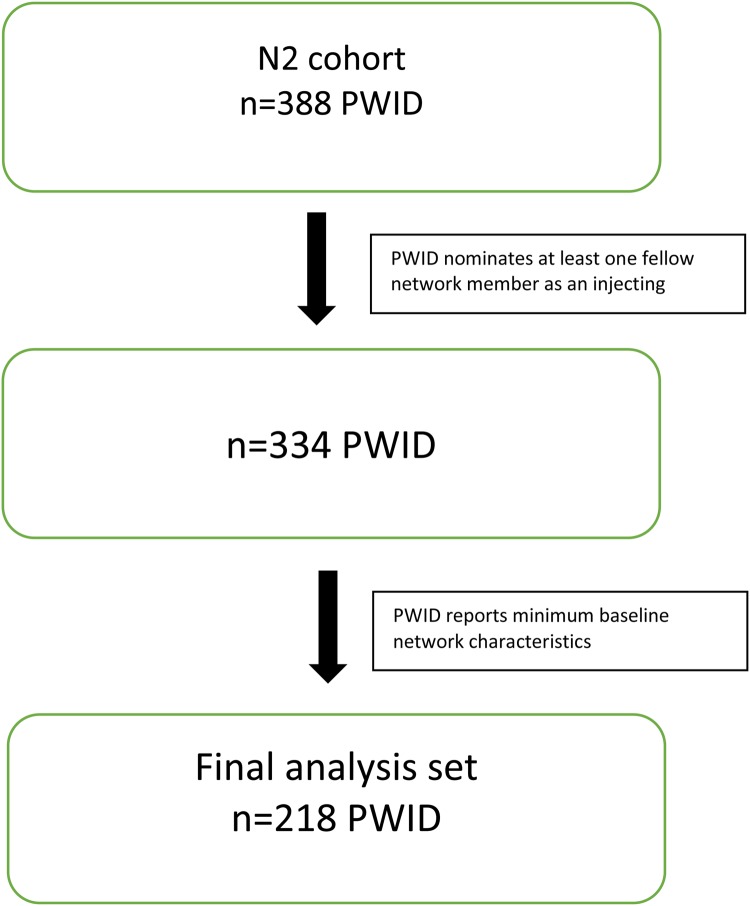
Summary of inclusions.

Participants for this analysis were sourced from the Networks 2 (N2) Study, a longitudinal, observational cohort study of PWID recruited from illicit drug street markets across Melbourne, Australia [35, 36]. Participants were interviewed and blood samples taken approximately every 3 months. Interviews included demography, injecting risk behaviours and their personal using and sharing network. The N2 dataset consisted of 388 PWID, contributing 1209 separate interviews between 12 July 2005 and 15 February 2010. From these 1209 interviews, there were 2456 reported ‘use with’ nominations. In order to contribute to the network analysis, PWID in the network cohort were required to nominate at least one fellow cohort member as an injecting partner. Furthermore, only injecting partners who were also participants of the cohort were included. Non-participating PWID who were nominated as injecting partners were excluded from the analysis. Of the total 2456 interviews in the starting sample, 998 (40.6%) nominated a PWID who was either not a fellow network member enrolled in the N2 study or who could not, from the information provided by the interviewee, be identified as an enrolled network member. Nominations where the nominee PWID could not be satisfactorily identified as a N2 network member were excluded. This resulted in a sample of 334 PWID contributing 931 interviews between 12 July 2005 and 15 February 2010, containing 1456 injecting partner nominations. The target assessment frequency across the larger N2 cohort was approximately 3 months for primary participants and annually for secondary participants. Of these, 218 reported the minimum level of baseline network characteristic data to be included in the analysis (Fig. 1). The mean (s.d.) number of interviews per network PWID was 2.79 (2.52) and the average number of nominations per interview was 1.56. The median (IQR) number of days between interviews was 114 (91, 203).

A ‘use with’ event was defined as the nominator PWID identifying a fellow PWID (the nominee) using in the same room or same place, in close proximity, at roughly the same time within the 3 months prior to the interview date. This includes injecting together but not necessarily sharing injecting equipment. Data on syringe sharing within ‘use with’ pairs was collected. Study recruitment employed a social networks approach, where previously enrolled PWID were requested to nominate up to a maximum of five of their most regular injecting partners. Recruited PWID were then asked to introduce study field workers to these partners to expand the sample explicitly along injecting relationship lines. Informed written consent was obtained for all recruited PWID and participation was voluntary. Ethics approval was obtained from the Victorian Department of Human Services Research Ethics Committee (project 02/05). All participants were offered both pre and post-test discussions for HCV, HBV and HIV. The study conformed to the ethical guidelines of the 1975 Declaration of Helsinki.

### Inclusions

PWID from the N2 study were included in this analysis if they contributed a minimum of two assessment points. Participants were required to contribute a minimum baseline assessment point of social networks data. Of the 388 PWID enrolled in the N2 study, 252 (62.5%) recorded a minimum of two assessment points. A total of 334 (86.1%) nominated at least one fellow network PWID as an injecting partner over the observation period. Of these, 218 (65.3%) recorded the required minimum baseline social networks data (Fig. 1).

### Social network

The social network is made up of a series of nodes (PWID) connected to other nodes by edges. An edge is defined as an injecting partner relationship within the recall period, as identified by a PWID at the interview. These relationships were modelled as undirected edges, in that either one or both members of a nominated injecting relationship may have nominated the other.

### Outcomes

Baseline was defined as the first recorded interview date in the study at which network structure data were also recorded for each PWID. The outcomes of this study were (1) time to injection frequency progression – first event and sustained progression; (2) time to injection frequency reduction – first event and sustained reduction and (3) change in injection frequency over time. Injecting frequency progression and reduction events were defined as an increase or decrease of at least one injection per month from baseline frequency, respectively, adjusting for baseline frequency. Sustained progression and reduction were defined as an increase or decrease in injection frequency sustained for at least 6 months at or above the previously recorded level. Change in injection frequency was defined as the number of injections recorded at a point in time minus the number of injections recorded at the previous assessment point.

### Network characteristics

A series of social network characteristics were explored as potential correlates. The primary explanatory social network characteristics flagged *a priori* as potentially correlates with risk progression and regression were degree, eccentricity, clustering coefficient, closeness centrality, betweenness centrality and eigenvector centrality (refer Table 1 for definitions). Each of these network characteristics was calculated for all individual PWID eligible for the analysis and modelled as both baseline and time-varying covariates.

**Table 1.** Description of injection drug network characteristics

Network metric	Definition	Network feature captured
Degree	In-degree + out-degree	Number of injecting partners
Eccentricity	Largest geodesic distance – the largest number of steps in the shortest possible walk from one PWID to every other PWID in the network	How far each PWID is from the furthest other in the network
Clustering coefficient	The ratio of actual edges between a PWID node and its neighbours to the total number of potential edges	The number of injecting partners relative to the total possible number of injecting partners
Closeness centrality	Inverse of the sum of distances to all other PWID	A measure of the centrality of a PWID within the injection drug network
Betweenness centrality	The number of shortest paths from all nodes to all other PWID that pass through a particular PWID node	The number of times a PWID acts as a bridge along the shortest path between two other PWID
Eigenvector centrality	How connected a PWID is those parts of the injecting network with the greatest connectivity	The extent to which ‘big fish’ connect with other ‘big fish’

### Statistical analyses

Categorical variables were summarised using frequency and percentage. Continuous variables were summarised using mean and standard deviation (s.d.) or median (IQR) as appropriate. Network predictors of injecting frequency progression and reduction were investigated using Cox Proportional Hazards models. As a sensitivity analysis, Generalised Estimating Equation regression modelling was used to model injection frequency as a continuous variable over time and the change from baseline in injection frequency. Each network metric was modelled as a predictor of each outcome adjusting for age, sex, baseline injection frequency, main drug used, OST and interview density. Interview density was defined as the number of assessment points contributed by an individual PWID as a proportion of their total follow-up duration. As PWID were able to contribute varying numbers of assessment points to the analysis, adjusting all models by interview density enabled control for systematic differences in progression and reduction event ascertainment opportunity. Hazard proportionality was assessed for Cox Proportional Hazards models through analysis of scaled Schoenfeld residuals. Hazard proportionality was satisfied for all models presented in this report. A Bayesian Information Criterion was used to assist in model selection. The linearity of association between candidate continuous explanatory variables and the various outcome variables were tested by incorporating quadratic transformations into the models. For each multivariate model, interactions between pairs of candidate predictors were further tested. All modelling was undertaken using R version 3.1.2 (R Foundation for Statistical Computing, Vienna, Austria).

## Results

### Participants

Two-hundred and eighteen PWID contributing 1385 assessment points were eligible for analysis. Median age at baseline was 25.5 years and males accounted for 143 (65.6%) of the analysis sample (Table 2). Most had evidence of current (56.0%) or past HCV infection. Median (IQR) age of first injection was 18 years (15, 20) and the median (IQR) duration of injecting career at baseline was 8 years (4.8, 11.6). Just over two-thirds were unemployed at baseline and less than a third reported unstable living arrangements. Approximately one-third reported receptive sharing of needles in the 3 months prior to baseline, and the median (IQR) number of times injected across the same period was 30 (12, 61) or approximately once every 3 days. Just over half the sample reported receiving opiate substitution therapy (OST) in the 3 months prior to baseline. Mean (s.d.) follow-up across the sample was 2.8 years (1.7) and the median (IQR) number of assessment points was 11.5 (6.5, 16.5).

**Table 2.** Baseline characteristics

Baseline characteristic	Level	Total (*n* = 218)
Gender – *n* (%)	Female	75 (34)
	Male	143 (65.6)
Age (years) – median (IQR)	–	25.5 (22.2–29.5)
Ethnicity – *n* (%)	Australian	146 (67)
	Vietnamese	34 (16)
	Other	38 (17)
Unemployed – *n* (%)	–	149 (68)
Stable living arrangements – *n* (%)	–	155 (71)
Age at first injection (years) – median (IQR)	–	18 (15, 20)
Injecting career (years) – median (IQR)	–	8 (4.8, 11.6)
Treatment ever (not limited to OST) – *n* (%)	–	186 (85)
OST in past 3 months – *n* (%)	–	113 (52)
Receptive sharing ever – *n* (%)	–	149 (68)
Prison ever – *n* (%)	–	77 (35)
Baseline HCV status – *n* (%)	Ab + , PCR + (current infection)	115 (53)
	Ab−, PCR + (seroconverting)	7 (3)
	Ab + . PCR− (past infection)	35 (16)
	Ab−, PCR− (never infected)	61 (28)
HIV infected – *n* (%)	–	2 (1)
HBV infection status – *n* (%)	Acute infection	1 (1)
	Chronic infection	7 (3)
	Prior infection	60 (28)
	Vaccinated	74 (34)
	Susceptible	51 (23)
	Unclear	25 (12)
Follow-up duration (years)	Mean (s.d.)	2.8 (2)
	
Baseline injection behaviour		
Times injected in last month – median (IQR)	–	30 (12–61)
Number of people used in the same room with in the past 3 months – median (IQR)	–	4 (2,7)
Per cent of the time used alone in past 3 months – median (IQR)	–	20 (0–50)
Receptive sharing in the past 3 months – *n* (%)	–	68 (31.2)
Baseline network characteristics	Summary measure	
Degree	Mean (s.d.)	3.4 (2.3)
	Median (IQR)	3 (2–5)
Eccentricity	Mean (s.d.)	2.9 (2.6)
	Median (IQR)	2 (1–4)
Closeness centrality	Mean (s.d.)	1.9 (1.4)
	Median (IQR)	1.4 (1–2.3)
Betweenness centrality	Mean (s.d.)	24.3 (58.9)
	Median (IQR)	0 (0–9.7)
Clustering coefficient	Mean (s.d.)	0.17 (0.28)
	Median (IQR)	0 (0, 0.25)
Eigenvector centrality	Mean (s.d.)	0.10 (0.19)
	Median (IQR)	0.02 (0.01–0.08)

PWID, people who inject drugs; OST, opiate substitution therapy; IQR, interquartile range; HCV, hepatitis C virus; HIV, human immunodeficiency virus; HBV, hepatitis B virus.

### Baseline network structure

Median (IQR) undirected number of injecting partners (degree) at baseline was 3 (2–5) (Table 2). Median (IQR) largest geodesic distance (eccentricity) was 2 (1–4) (Table 1). The mean (s.d.) baseline ratio of observed to potential edges per PWID node (clustering coefficient) was 0.17 (0.28).

Median (IQR) closeness centrality at baseline was 1.4 (1–2.3) whilst mean (s.d.) eigenvector centrality was 0.10 (0.19).

### Increased injection frequency

Across the observation period, 162 PWID increased their injection frequency at least once. Of these, in 75 (46.3%) this higher rate of injection was sustained for at least 6 months. Reporting at least one partner PWID at baseline was associated with 1.08 times the rate of increased injection frequency events (adjusted hazard ratio (aHR) 1.08; 95% CI 1.02–1.18) adjusting for age, sex, baseline injection frequency, main drug used, OST and interview density (Table 3). Whilst every additional injecting partner at baseline did not correlate when modelled as a continuous variable, the same predictor was associated with a 10% reduction in the rate of 6-month sustained injecting frequency (aHR 0.90; 95% CI 0.81–0.99).

**Table 3.** Cox proportional hazards model: baseline and time-varying network metrics as predictors of injecting frequency progression

		First progression event (events = 162)	Six-month sustained progression (events = 75)
Baseline network metric	Level	Adjusted HR (95% CI) *P*-value^a^	Adjusted HR (95% CI) *P*-value^a^
Degrees	Continuous	1.00 (0.94–1.07) 0.966	**0.90 (0.81–0.99) 0.044**
	1 + degrees	**1.08 (1.02–1.18) 0.032**	0.54 (0.22–1.33) 0.181
Eccentricity	Continuous	0.95 (0.89–1.02) 0.130	1.02 (0.93–1.11) 0.666
	1+	**0.49 (0.28, 0.85) 0.012**	0.80 (0.36, 1.77) 0.588
	2+	**0.64 (0.46, 0.89) 0.009**	0.72 (0.45, 1.17) 0.184
Closeness centrality	Continuous	**0.88 (0.74–0.98) 0.039**	1.04 (0.88–1.23) 0.670
Betweenness centrality	Continuous	1.00 (1.00–1.01) 0.415	1.00 (0.99–1.00) 0.671
Clustering coefficient	Continuous	0.83 (0.46–1.47) 0.514	0.47 (0.17–1.29) 0.144
Eigenvector centrality	Continuous	0.59 (0.28–1.26) 0.172	0.36 (0.09–1.41) 0.142
Time-varying network metric	Level	Adjusted HR (95% CI) *P*-value^b^	Adjusted HR (95% CI) *P*-value^b^
Degrees	Continuous	1.04 (0.99–1.10) 0.151	1.04 (0.78–1.28) 0.828
	1 + degrees	**1.41 (1.21–1.64) <0.001**	1.05 (0.86–1.20) 0.479
Eccentricity	Continuous	0.99 (0.75–1.30) 0.923	**0.78 (0.56–0.99) 0.048**
	1+	0.95 (0.80, 1.14) 0.600	**0.73 (0.57, 0.95) 0.017**
	2+	**0.47 (0.39, 0.56) <0.001**	0.84 (0.65, 1.09) 0.190
Closeness centrality	Continuous	**1.32 (1.19–1.46) <0.001**	**1.25 (1.01–1.54) 0.043**
Betweenness centrality	Continuous	1.01 (0.98–1.03) 0.560	0.99 (0.96–1.03) 0.617
Clustering coefficient	Continuous	0.78 (0.55–1.10) 0.158	0.67 (0.39–1.15) 0.143
Eigenvector centrality	Continuous	**1.40 (1.20–1.63) <0.001**	**1.25 (1.02–1.55) 0.039**

a Each network metric modelled separately adjusted for sex, baseline injection frequency and interview density.

b Each network metric modelled separately adjusted for age, sex, baseline injection frequency and interview density.

PWID with fewer injecting partners at baseline tended to be located out towards the network periphery and were associated with a decreased rate of injection frequency (1+ eccentricity aHR 0.49; 95% CI 0.28–0.85), relative to more central PWID reporting a larger number of injecting partners. Densely-connected PWID with comparatively large numbers of injecting partners, who were themselves linked to other densely connected nodes were, on average, associated with 1.40 times the rate of first progression event (eigenvector centrality aHR 1.40; 95% CI 1.20–1.33), although this relationship did not translate into a sustained increase in injection frequency at 6 months. Whilst an increase in clustering of PWID did not predict a changed rate of the injection frequency increase, it did correlate with a *reduction* in the rate of sustained increase at 6 months (aHR 0.46; 95% CI 0.27–0.77).

When these explanatory network characteristics were modelled as time-varying factors, a minimum of one extra injection partner over time was again associated with an increase in injection frequency (aHR 1.41; 95% CI 1.21–1.64). Whilst increasing the number of injecting partners over time (i.e. increased node degree) predicted an increase in injection frequency, this was not sustained for more than 6 months. Those PWID who migrated from the network centre and towards the periphery over time (e.g. PWID who decreased the number of injecting partners over the observation period) were associated with a 53% reduction in injection frequency (aHR 0.47; 95% CI 0.39–0.56). Furthermore, as the overall size of injection drug network increased (i.e. an increase in geodesic step), the rate of 6-month sustained increase in injection frequency decreased (aHR 0.78; 95% CI 0.56–0.99).

Subsets of PWID who increased their injection frequency from baseline levels became more central in the network and more densely connected over the observation period. Increase closeness centrality was associated with both 1.32 times the rate of injection frequency (aHR 1.32; 95% CI 1.19–1.46) and 6-month sustained increase in monthly injection frequency (aHR 1.25; 95% CI 1.01–1.54). Similarly, increased network eigenvector centrality was also associated with an increased rate of injection frequency (aHR 1.40; 95% CI 1.20–1.63) and 6-month sustained increase (aHR 1.25; 1.02–1.55).

As a sensitivity analysis, we further modelled the association between increasing the number of injecting partners and different quantities of injection frequency change. Every additional injecting partner was associated with 1.09 times the rate of a minimum injecting frequency increase of 10 (HR 1.09; 95% CI 1.01–1.26). Similarly, every extra injecting partner was associated with 1.17 times the rate of at least 20 additional injections (HR 1.17; 95% CI 1.01–1.34).

### Injection frequency reduction

Relative to observed increase in injection frequency events, injection frequency reduction events were more frequent (218 events) (Table 4). However, a comparatively smaller proportion of these were sustained for 6 months (43, 19.7%). Interestingly, an increasing number of injecting partners at baseline was associated with a reduction in injection frequency (aHR 1.09, 95% CI 1.02–1.16), commonly following an initial increase in injection frequency. The tendency of highly-connected PWID with lots of injecting partners to inject with other highly connected nodes was similarly correlated with an increased rate of reduction (aHR 1.47; 95% CI 1.01–2.24), although this reduction typically followed a preceding increase in injection frequency. Furthermore, this reduction was rarely sustained over 6 months

**Table 4.** Cox proportional hazards model: baseline and time-varying network metrics as predictors of injecting frequency reduction

		First reduction event (events = 218)	Six-month sustained reduction (*n* = 43 events)
	Level	Adjusted HR (95% CI) *P*-value^a^	Adjusted HR (95% CI) *P*-value^b^
Baseline network metric			
Degrees	Continuous	**1.07 (1.01–1.14) 0.043**	1.10 (0.95–1.27) 0.191
	1 + degrees	0.95 (0.47–1.95) 0.898	1.50 (0.20–11.08) 0.688
Eccentricity	Continuous	0.97 (0.91–1.02) 0.219	0.91 (0.80–1.04) 0.162
	1+	0.96 (0.56, 1.64) 0.877	0.89 (0.31, 2.51) 0.891
	2+	0.84 (0.62, 1.14) 0.268	0.70 (0.38, 1.31) 0.267
Closeness centrality	Continuous	0.94 (0.85–1.05) 0.258	0.87 (0.69–1.10) 0.232
Betweenness centrality	Continuous	1.00 (0.99–1.00) 0.635	1.00 (0.99–1.01) 0.936
Clustering coefficient	Continuous	0.99 (0.59–1.68) 0.997	1.20 (0.40–3.56) 0.742
Eigenvector centrality	Continuous	1.53 (0.72–3.25) 0.269	0.93 (0.14–6.37) 0.941
	>0	**1.47 (1.01–2.24) 0.041**	1.85 (0.77–4.41) 0.168
Time-varying network metric			
Degrees	Continuous	**1.10 (1.05–1.16) <0.001**	**1.14 (1.00–1.29) 0.048**
	1 + degrees	1.47 (0.85–2.54) 0.173	1.93 (0.48–7.81) 0.355
Eccentricity	Continuous	**1.21 (1.05–1.69) 0.001**	**1.25 (1.11–1.41) <0.001**
	1+	**2.41 (2.02, 2.86) <0.001**	**3.34 (2.27, 4.92) <0.001**
	2+	**1.21 (1.01, 1.44) 0.036**	1.28 (0.87, 1.90) 0.211
Closeness centrality	Continuous	0.89 (0.58–1.36) 0.591	1.19 (0.52–2.73) 0.683
Betweenness centrality	Continuous	1.00 (0.98–1.02) 0.842	1.01 (0.97–1.06) 0.542
Clustering coefficient	Continuous	1.06 (0.75–1.49) 0.733	1.06 (0.47–2.38) 0.882
Eigenvector centrality	Continuous	1.28 (0.85–1.91) 0.240	0.79 (0.25–2.53) 0.693
	>0	0.92 (0.71–1.20) 0.557	1.16 (0.72–1.85) 0.542

a Each network metric modelled separately adjusted for sex, baseline injection frequency and interview density.

b Each network metric modelled separately adjusted for age, sex, baseline injection frequency and interview density.

Comparatively stronger associations were observed within the time-varying network metrics. Additional injecting partners over the observation period were associated with an increase in injection frequency reduction events (aHR 1.10; 95% CI 1.05–1.16). However, in contrast to the baseline modelling, this increase in the rate of the initial reduction event was sustained over 6 months of observation. Whilst this result was unexpected, the movement of PWID over time from central network positions towards the periphery (e.g. by reporting fewer and fewer injecting partners over time) was also associated with an increase in the rate of injection frequency reduction, both the initial observed event (aHR 1.21; 95% CI 1.05–1.69) and subsequent 6-month regression (aHR 1.25; 95% CI 1.11–1.41).

### Change in injection frequency from baseline

There was no association between a change in injection frequency from reported baseline frequency and an increase in the number of injecting partners (*β* −0.28; 95% CI −0.95 to 0.38) (Table 5). PWID reporting fewer injecting partners and located further out in the network periphery demonstrated increasingly larger reductions in injection frequency, relative to more centrally located PWID, with every additional step away from the network centre associated with, on average, 1.67 less injections per month (*β* = −1.67; 95% CI −2.32 to −1.04). This reduction from baseline injecting frequency was even observed at relatively short distances away from the centre with location of *at least* one step away from the centre associated with 22.93 less injections per month (*β* −22.93; 95% CI −29.22 to −16.63), relative to an eccentricity of zero. This suggests that even modest reductions in the number of injecting partners may be associated with meaningful reductions in injection frequency.

**Table 5.** Generalised Estimating Equation models of associations between baseline and time-varying network characteristics with change from baseline in monthly injection frequency

Baseline network metric	Level	Adjusted *β* coefficient (95% CI) *P*-value^a^
Degrees	Continuous	−0.28 (−0.95 to 0.38) 0.406
	1 + degrees	**−25.50 (−35.34 to −15.65) <0.001**
Eccentricity	Continuous	**−1.67 (−2.31 to −1.04) <0.001**
	1 +	**−22.93 (−29.22 to −16.63) <0.001**
	2 +	**−6.01 (−9.66 to 2.36) 0.001**
Closeness centrality	Continuous	**5.41 (4.13–6.70) <0.001**
Betweenness centrality	Continuous	**0.05 (0.03–0.07) <0.001**
Clustering coefficient	Continuous	−2.89 (−10.14 to 4.35) 0.433
	>0	**8.05 (4.46–11.65) <0.001**
Eigenvector centrality	Continuous	**9.93 (2.15–17.70) 0.012**
Time-varying network metric		
Degrees	Continuous	**2.42 (1.18–3.66) <0.001**
	1 + degrees	−1.64 (−21.46 to 18.18) 0.871
Eccentricity	Continuous	1.42 (−0.39 to 3.24) 0.124
	1 +	**−13.76 (−18.57 to −8.95) <0.001**
	2 +	**−5.61 (−9.54 to −1.68) 0.005**
Closeness centrality	Continuous	−1.85 (−4.93 to 1.23) 0.239
Betweenness centrality	Continuous	**1.30 (0.90–1.70) <0.001**
Clustering coefficient	Continuous	4.52 (−4.33 to 13.36) 0.317
	>0	**8.85 (4.50–13.19) <0.001**
Eigenvector centrality	Continuous	**15.35 (7.10–23.61) <0.001**

Bold values are statistically significant (*p*-value >0.5).

a Each network metric modelled separately adjusted for age, sex, main drug, OST and interview density

Increased clustering of PWID within the network was associated with an increase in injection frequency, with a non-zero clustering coefficient associated with, on average, an extra 8.05 injections per month over reported baseline frequency (*β* = 8.05; 95% CI 4.46–11.65). Similarly, as average baseline network connectivity increases (i.e. increased node eigenvector centrality), so too does post-baseline injecting frequency, with increasing eigenvector centrality associated with a 9.93 additional monthly injections over baseline levels (*β* = 9.93; 95% CI 2.15–17.70). Further, an increase in the number of shortest paths passing through any one PWID was associated with a marginal increase in injection frequency (betweenness centrality *β* = 0.05; 95% CI 0.03–0.07). Consistent with these observations, increasing baseline closeness centrality was also associated with an increase in injection frequency over time (*β* = 5.41; 95% CI 4.13–6.70). These correlations were observed both within the baseline network *and* across time (Table 5).

Every additional injecting partner at baseline was further associated with 1.04 times the rate of incident HCV infection (adjusted HR 1.04; 95% CI 1.01–1.09) (Supplementary Table S1). Similarly, every additional injecting partner over the observation period was associated with 1.05 times the rate of HCV infection (adjusted HR 1.05; 95% CI 1.02–1.13).

## Discussion

Social network characteristics of PWID have previously been flagged as potential risk factors for HCV transmission. However, most studies have focused on HIV risk networks. This analysis of a longitudinal injection drug network of PWID observed that structure and arrangement of interactions between PWID within a risk network, both at baseline and across time, may predict fluctuations in injection frequency and, by extension, HCV transmission risk. Indeed, this observation may be extended to other blood-borne viruses including HIV.

Having at least one injecting partner and increasing eigenvector centrality at baseline were associated with an increased rate of injection frequency, whilst larger baseline eccentricity was associated with a reduction. Increasing both the number of injecting partners, closeness centrality and eigenvector centrality over time were similarly associated with an increased rate of injection frequency. These results suggest that PWID who decrease their number of injecting partners over time (i.e. move from the network centre and towards the periphery) were less likely to report an increase in injection frequency, relative to PWID placed more centrally within the network. These increased injection frequency events however appeared short-lived with just under half of the first increased injecting frequency events (75, 46.3%) being sustained for a minimum of 6 months and only 33 (20.4%) being sustained for at least 12 months.

Increasing the number of injecting partners over time was also associated with a reduced rate of injection frequency, both in terms of any reduction event and 6- and 12-month sustained reduction. This observation that an increase in the number of injecting partners was associated with both an initial increase in injection frequency often followed by a significant reduction appeared to be localised more towards the centre of the network where the number of injecting partners was highest. In other words, PWID positioned more centrally within the risk network and closer to the more densely connected sub-populations were far more volatile in their injection frequency habits, recording more frequent increases and decreases in injection frequency, relative to PWID who reduced their number of injection partners. Furthermore, this subset of PWID more frequently reported moving in and out of OST. Whilst OST itself did not statistically significantly correlated with injection frequency, this may in part influence the observation that more densely connected PWID tended to first increase, and then significantly decrease, their injection frequency, as has been previously observed [37].

This observation that moving from central, densely connected locations to more peripheral, less connected positions across the observation period was associated with, on average, a decrease in injection frequency, was further supported by the observed correlation between increasing overall network size (eccentricity) and outcome. Furthermore, increased centrality over time, as measured by both closeness and eigenvector centrality as time-varying factors, was associated with a 6-month sustained increase in injection frequency modelled as a categorical event outcome and an overall significant increase in the number of additional injections per month.

This correlation between a change in network position and injection frequency has potentially important implications. By reflecting changes in both injection frequency and the number of injecting partners, changes over time in a PWID's position within a network may potentially act as a proxy for HCV transmission risk. However, the relatively small effect size observed in our incident HCV modelling (i.e. every additional injecting partner was associated with 1.04 times the rate of HCV infection; *P* = 0.048) is unlikely to be clinically significant. Thus, our present results do not provide strong evidence for using injection frequency as a transmission proxy. A larger sample with longer follow-up would be required to both better characterise this correlation and to formally test its utility as a proxy for transmission. This in turn has implications for the design and implementation of risk reduction strategies and targeting the roll-out of new era direct acting antivirals (DAAs). Overall, our results suggest the presence of a stable network periphery surrounding a relatively changeable inner core. This means that moving away from the network core, by reducing the number of injection partners, may result in a sustained reduction of injection frequency. This finding potentially has ramifications for the rolling out of DAAs, as well as harm reduction and prevention measures. These results suggest that treatment efforts could be preferentially directed towards the volatile inner core of the network to potentially maximise impact.

This study has a number of limitations. Not all of the enrolled PWID in the Networks 2 were able to be included in the analysis set secondary to uncertainty around the identity of a subset of PWID nominated as injecting partners by study participants, despite exhaustive efforts by field and office staff to confirm identities and attempts at statistical probabilistic matching. The data collection also relies on self-report and thus is prone to be associated with recall bias. There was also considerable variation in the median time between assessments between PWID. Given the difficulties involved in following PWID longitudinally, both duration and frequency of follow-up interviews were inconsistent across participants. These issues were in part managed in the analysis by adjusting both the time-to-event and change in injection frequency models by interview density – the number of assessment points divided by the years of follow-up at the level of each participating PWID. The popularity of heroin may also have limited the ability to investigate the influence of other injectables due to underpowering. Overdose data were not collected and thus could not be included in the analysis. This study does have a number of strengths and advantages over previous, comparable studies. Most notably the longitudinal nature of the data permitted an analysis of how the *change* in network characteristics over time impacted on injection frequency. This is in contrast with much of the available HCV networks literature which focuses on cross-sectional networks, typically at a single point in time (i.e. baseline) only.

## Conclusion

This study corroborates previous observations that baseline network measures of connectivity, centrality and dispersion may be associated with changes in injection frequency and, by extension, possibly predict subsequent HCV transmission risk. It extends these previous observations to include longitudinal changes in network position as correlates of injection frequency – with PWID who decrease their number of injection partners associated with a decreased rate of injection frequency. This underscores the importance of regular testing for enabling PWID to be informed on the HCV status of injecting partners. It further supports maintaining a low threshold for entry into OST for PWID wishing to reduce their injection frequency and transmission risk.

## Supplementary material

For supplementary material accompanying this paper visit https://doi.org/10.1017/S095026881900061X. click here to view supplementary material
