# Supplementary material for: Injection drug network characteristics as a predictor of injection behaviour
Source: Epidemiol Infect. 2019 Apr 5;147:e173. doi: 10.1017/S095026881900061X (PMC6518653; doi:10.1017/S095026881900061X)
Supplement: Supplementary file 1 [file S095026881900061Xsup001.docx]

**Supplementary Table 1 – Cox Proportional Hazards model of associations between baseline and time-varying network characteristics with time to incident HCV infection**

|  | | **Time to incident HCV infection - Cox PH** |
| --- | --- | --- |
| **Baseline network metric** | **Level** | **Adjusted HR (95% CI) p-value^^^** |
| Degrees | Continuous | **1.04 (1.01, 1.09) 0.048** |
|  | 1+ degrees | 1.61 (0.22, 11.78) 0.639 |
|  | 2+ degrees | 1.31 (0.55, 3.17) 0.542 |
|  | 3+ degrees | 1.23 (0.62, 2.42) 0.552 |
| Eccentricity | Continuous | 1.02 (0.90, 1.15) 0.766 |
|  | 1+ | 1.03 (0.32, 3.37) 0.960 |
|  | 2+ | 1.09 (0.54, 2.17) 0.816 |
|  | 3+ | 0.90 (0.45, 1.78) 0.761 |
| Closeness Centrality | Continuous | 1.05 (0.83, 1.34) 0.658 |
| Betweeness Centrality | Continuous | 1.00 (0.99, 1.00) 0.462 |
| Clustering Coefficient | Continuous | 0.36 (0.07, 1.80) 0.216 |
| Eigenvector Centrality | Continuous | 0.37 (0.05, 2.67) 0.324 |
| **Time varying network metric** |  | **Adjusted HR (95% CI) p-value^^^** |
| Degrees | Continuous | **1.05 (1.02, 1.13) 0.042** |
|  | 1+ degrees | 1.39 (0.33, 5.78) 0.654 |
|  | 2+ degrees | 1.05 (0.54, 2.04) 0.887 |
|  | 3+ degrees | 0.78 (0.30, 2.01) 0.606 |
| Eccentricity | Continuous | 0.97 (0.71, 1.33) 0.841 |
|  | 1+ | 1.35 (0.61, 2.97) 0.462 |
|  | 2+ | 1.10 (0.53, 2.30) 0.790 |
|  | 3+ | 0.52 (0.12, 2.18) 0.372 |
| Closeness Centrality | Continuous | 1.00 (0.61, 1.63) 0.996 |
| Betweeness Centrality | Continuous | 0.77 (0.51, 1.16) 0.210 |
| Clustering Coefficient | Continuous | 0.64 (0.09, 4.77) 0.667 |
| Eigenvector Centrality | Continuous | 1.90 (0.33, 10.77) 0.471 |
| ^Each network metric modelled separately adjusted for age, sex and interview density; all models satisfied hazard proportionality | | |
